# The Impact of a Quinone Scaffold on Thermo-TRPs Modulation by Dimethylheptyl Phytocannabinoids

**DOI:** 10.3390/ijms26062682

**Published:** 2025-03-17

**Authors:** Aniello Schiano Moriello, Aurora Bossoni, Daiana Mattoteia, Diego Caprioglio, Alberto Minassi, Giovanni Appendino, Luciano De Petrocellis, Pietro Amodeo, Rosa Maria Vitale

**Affiliations:** 1 Institute of Biomolecular Chemistry, National Research Council (ICB-CNR), Via Campi Flegrei 34, 80078 Pozzuoli, NA, Italy; aniello.schianomoriello@icb.cnr.it (A.S.M.); luciano.depetrocellis@icb.cnr.it (L.D.P.); pamodeo@icb.cnr.it (P.A.); 2 Epitech Group SpA, Via Leonardo Da Vinci 3, 35030 Saccolongo, PD, Italy; 3 Dipartimento di Scienze del Farmaco, Università del Piemonte Orientale, Largo Donegani 2, 28100 Novara, NO, Italy; aurora.bossoni@unipo.it (A.B.); daiana.mattoteia@uniupo.it (D.M.); diego.caprioglio@unipo.it (D.C.); alberto.minassi@unipo.it (A.M.); giovanni.appendino@unipo.it (G.A.)

**Keywords:** thermo-TRPs, phytoquinones, molecular docking, synthesis, fluorescence-based assay

## Abstract

Phytocannabinoids (pCBs) from *Cannabis sativa* represent an important class of bioactive molecules, potentially useful for the treatment of a wide range of diseases. Their efficacy is due to their ability to interact with multiple targets of the endocannabinoid system, including the thermosensitive transient receptor potential (Thermo-TRPs), namely TRPV1-4, TRPA1, and TRPM8 channels. Previously, we demonstrated a shift in selectivity toward TRPA1 in the activity profile of the main pCBs, that is, CBD, ∆^8^-THC, CBG, CBC, and CBN, by swapping the pentyl chain with the α,α-dimethylheptyl (DMH) one. Using these derivatives as a starting point, here we investigate the effects on the thermo-TRPs activity profile of the integration of a quinone group into the resorcinol scaffold. We found that, while the activity on TRPA1 is substantially retained, an increase in potency/efficacy on the TRPV3 modulation is observed. Docking studies were used to elucidate the binding modes of the most active compounds toward this receptor, providing a rationale for this biological activity. In summary, we show that the quinone derivatives of DMH-pCBs are endowed with a TRPA1/TRPV3 desensitizing activity, potentially useful for the treatment of skin diseases sustained by inflammatory conditions.

## 1. Introduction

Phytocannabinoids (pCBs) are a class of bioactive compounds from *Cannabis sativa*, a plant traditionally used for medicinal and recreational purposes. To date, more than 150 pCBs have been characterized, although for most of them the pharmacological potential has been only marginally exploited, due to their natural low abundance [1,2]. From a structural point of view, pCBs are meroterpenoids, characterized by an aromatic core, resorcinol, linked to a *para*-oriented alkyl linear chain and an isoprenyl substituent. The high structural diversity of the pCBs chemotype mainly arises from the combinatorial tunability of these two kinds of substituents. In fact, the alkyl chain can span from one to five carbon atoms, although the pentyl chain is far more common, while the isoprenyl moiety can adopt linear, mono- or even poly-cyclic structures. The combination of an aromatic scaffold and both polar and hydrophobic functional groups renders these compounds able to interact with a wide range of molecular targets, ranging from enzymes to receptorial systems, thus eventually exhibiting complex biological profiles typical of multitarget agents [3,4,5]. The pharmacological relevance of the most investigated pCBs, such as ∆^9^-tetrahydrocannabinol (∆^9^-THC) and cannabidiol (CBD) [6,7], is mainly due to their ability to interact with and modulate the endocannabinoid system, which also include, besides the cannabinoid receptors CB1 and CB2, a subset of the transient receptor potential (TRP) channel superfamily, known as “thermosensitive transient receptor potential” (thermo-TRPs) channels, also referred to as ionotropic cannabinoid receptors [8,9]. TRPs are transmembrane ligand-gated cation channels, widely expressed in neuronal and non-neuronal tissues, where they act as polymodal sensors for both chemical and physical stimuli. TRP members mediate distinct physiological functions, ranging from thermosensation, mechanosensation and taste perception, to homeostasis, osmolarity, cardiovascular functions, cell proliferation, up to inflammation, and nociception [10,11,12]. The functional unit of TRPs is a tetramer, where each monomer, assembled around a central permeation pore, features a N- and C-terminal cytosolic domain and a transmembrane (TM) region. The latter is organized in six helices (S1–S6) and a C-terminal amphipathic helix called TRP domain, parallelly oriented to the membrane plane. The S1–S6 region, in turn, includes a S1–S4 helix bundle arranged in a voltage-sensor like domain (VSLD) and a channel pore formed by segment S5, a pore loop/helix and segment S6 [13,14]. According to their sequence homology, TRPs are grouped in six sub-families: TRPV, TRPA, TRPM, TRPP, TRPML, and TRPN [15]. TRPV1-4, TRPA1, and TRPM8 are also referred to as thermo-TRPs, due to their role in the transduction of thermosensation and thermal nociception [16,17,18,19]. TRPV1, the first discovered member of the TRPV subfamily, is activated by capsaicin, the bioactive ingredient of hot chili peppers, responsible for the pungency and the burnt pain sensation of this spice. TRPV1, which is predominantly expressed in nociceptive neurons, plays a key role in pain and neurogenic inflammation [20]. TRPV2 is widely expressed in the tissue and organs, where it is involved in calcium homeostasis. It is also involved in cardiomyopathies [21] and in degenerative muscular diseases [22], as well as in cancer metastasis [23]. TRPV3 is mainly expressed in skin keratinocytes, where it contributes to the maintenance of the skin barrier, epidermal proliferation and hair growth and mediates thermal sensation, cutaneous nociception, and itch [24,25]. TRPV3 dysfunctions are responsible for skin diseases such as atopic dermatitis and human congenital skin disorder such as the Olmsted syndrome [26,27]. TRPV3 is activated by camphor, the bioactive ingredient of topical analgesics [28]. TRPV4 is widely expressed in human tissues, where it is involved in nociception, osmotic regulation, endothelial function, epithelial ciliary activity, and in pathological processes such as inflammatory lung diseases [29]. TRPA1, activated by pungent compounds [30] occurring, for example, in mustard, ginger, and garlic, plays a key role in neuropathic and inflammatory pain, representing a pharmacological target for analgesic drugs [31,32]. The TRPM8 channel is activated by low temperature and cooling agents, such as menthol and icilin, and mediates both the analgesic effects of menthol and cold hyperalgesia [33]. In addition to sensory neurons, TRPM8 is also expressed in a range of tissues, including the prostate gland, where it is involved in urinary bladder disorders and in cancer [34].

Given the pharmacological relevance of pCBs as modulators of the endocannabinoid system, many synthetic efforts have been devoted to modifying the natural scaffold, with the aim of increasing the potency and/or the selectivity on specific molecular targets. For example, the occurrence of the α,α-dimethylheptyl (DMH) in place of the *n*-pentyl chain, as in the synthetic cannabinoids CP-55,940 and HU-210, led to a dramatic increase in binding affinity toward the cannabinoid receptors and also conferred partial agonist/negative allosteric activity to CBD toward CB2R [35]. Moreover, we have recently shown that the *n*-pentyl → DMH replacement on the five major phytocannabinoids [CBD (**5a**), ∆^8^-THC (**6a**), CBG (**7a**), CBC (**8a**), and CBN (**9a**)] promoted a shift in the selectivity toward TRPA1 within the panel of thermo-TRPs [36]. On the other hand, quinones represent a large and variegate class of bioactive compounds [37,38]. For example, anthracyclines are approved drugs as antibiotic and/or anticancer agents, although their use is limited by cumulative cardiac toxicity [39]. As it concerns phytocannabinoids, the first report of a cannabinoid quinone was the compound HU-331, synthetized by the Mechoulam group in 1968. This compound is highly effective as an anticancer agent in in vitro and in vivo mouse models and, in contrast to other quinones, devoid of any cardiotoxicity [40]. Apart the anticancer activity, many other biological effects have been reported for cannabinoid quinones so far [37]. For example, the methoxylated derivative of HU-331 has been shown to be neuroprotective in in vitro models of Huntington’s disease due to its anti-inflammatory and antioxidant properties [41].

Thus, in continuing our research in the field, we decided to further modify the DMH-pCB derivatives by integrating the quinone group on the resorcinol scaffold and to evaluate its effect on the modulation of thermo-TRPs of this class of pCBs.

## 2. Results

### 2.1. Synthesis

**Scheme 1:**
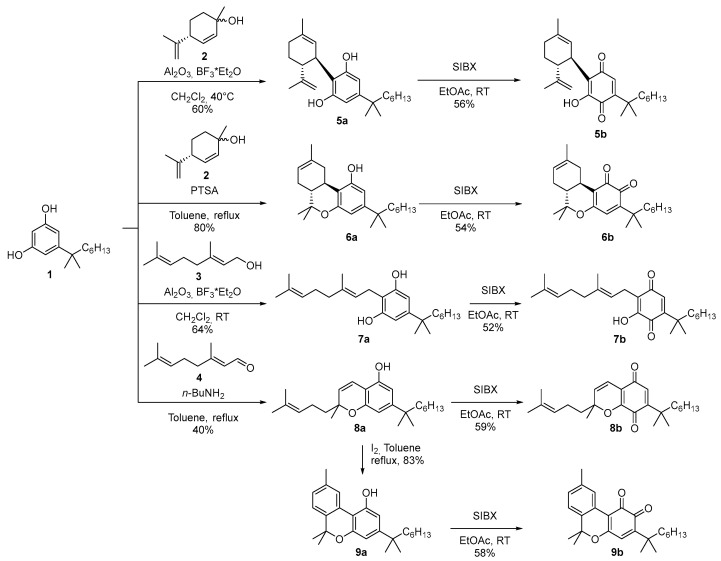
Synthesis of α,α-dimethylheptyl quinone analogs of major phytocannabinoids.

α,α-DMH-quinone analogs of the major phytocannabinoids were synthesized via stabilized 2-Iodoxybenzoic acid (SIBX) oxidation of their unoxidized precursors, which were derived from α,α-DMH-resorcinol (**1**), itself prepared from dimethoxybenzoic acid as a starting material [36,42]. The reaction of **1** with (4R)-2,9-*p*-menthadien-1-ol (**2**) under C-menthylation conditions led to the selective formation of either DMH-CBD (**5a**) or DMH-Δ⁸-THC (**6a**), depending on the specific reaction parameters employed [43]. Alkylation with geraniol (**3**), catalyzed by BF_3_ supported on alumina, produced DMH-CBG (**7a**) [44]. Chromenylation using citral (**4**) under basic conditions resulted in DMH-CBC (**8a**) [45], which was subsequently converted into DMH-CBN (**9a**) through an iodine-mediated aromatization step (Scheme 1).

The obtained α,α-DMH-cannabinoids (**5a**, **6a**, **7a**, **8a**, and **9a**) were then oxidized using SIBX following literature-reported protocols to obtain the corresponding *o*-quinones (**6b** and **9b**, respectively) or *p*-quinones (**5b**, **7b** and **8b**, respectively) [46] (Scheme 1). Unlike the *o*-quinones derived from *n*-pentyl cannabinoids, the *o*-quinones of the α,α-DMH analogs exhibited remarkable stability, even at room temperature. This enhanced stability is likely attributable to the presence of the geminal dimethyl group in the benzylic position, which, through steric hindrance, may inhibit the reactivity of the electrophilic site on the *o*-quinone, thereby reducing its susceptibility to further degradations.

### 2.2. Biological Evaluation

The activity profile at the thermoTRPs, namely TRPV1-4, TRPA1, and TRPM8, of the quinone derivatives **5b**–**9b**, is reported in Table 1 and Table 2. All the compounds act as potent agonists/desensitizers at TRPA1 in a low micromolar range except for compound **9b**, with compound **8b** being the most active with an EC_50_/IC_50_ value < 1 μM. A submicromolar potency as agonists/desensitizers was shown at TRPV3 by both **5b** and **6b**. The rank of inhibitory potency within the series is **6b** (IC_50_ = 0.72 μM)~**5b** (IC_50_ = 0.76 μM) > **7b** (IC_50_ = 8.6 μM) > **9b** (IC_50_ = 19.4 μM) > 8b (IC_50_ = 38.3 μM). At TRPV2, all the compounds behave as antagonists with an IC_50_ ranging from ~5 to ~8 μM except for **7b** (IC_50_ = 23.7 μM). Instead, all the investigated compounds are inactive at both TRPV1 and TRPV4 (see Table 1), while at TRPM8 only compounds **5b** and **7b** are active as antagonists, with compound **5b** performing better than **7b** (1.2 vs. 14.9 μM).

### 2.3. Molecular Docking Studies on Compound 5b and 6b at TRPV3 Channel

**Figure 1:**
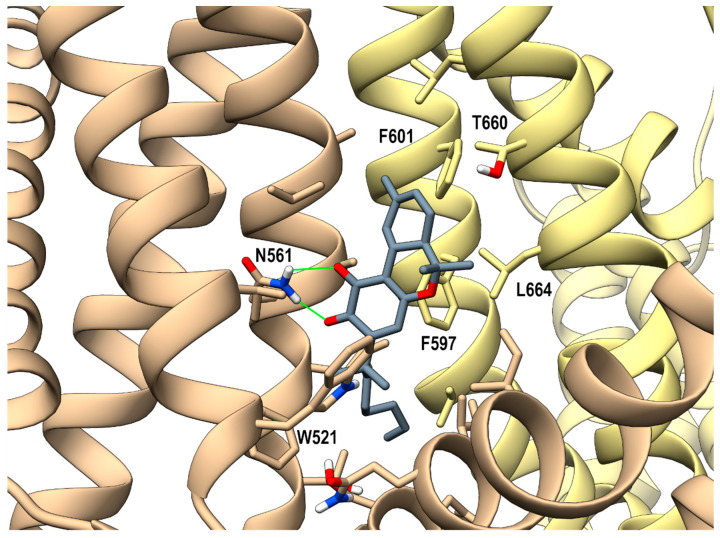
hTRPV3 in a complex with compound **6b** (p-sI-A) colored in dark gray at the vanilloid site (sI in the main text). A ribbon representation is used for the protein backbone and sticks for protein side chains of residues within 5 Å from the ligand, in a ball and stick representation. The two adjacent monomers are colored in tan and yellow. Carbon atoms are painted according to receptor subunits. H-bonds are shown as green sticks. Nitrogen, oxygen, and polar hydrogen atoms are painted blue, red, and white, respectively.

**Figure 2:**
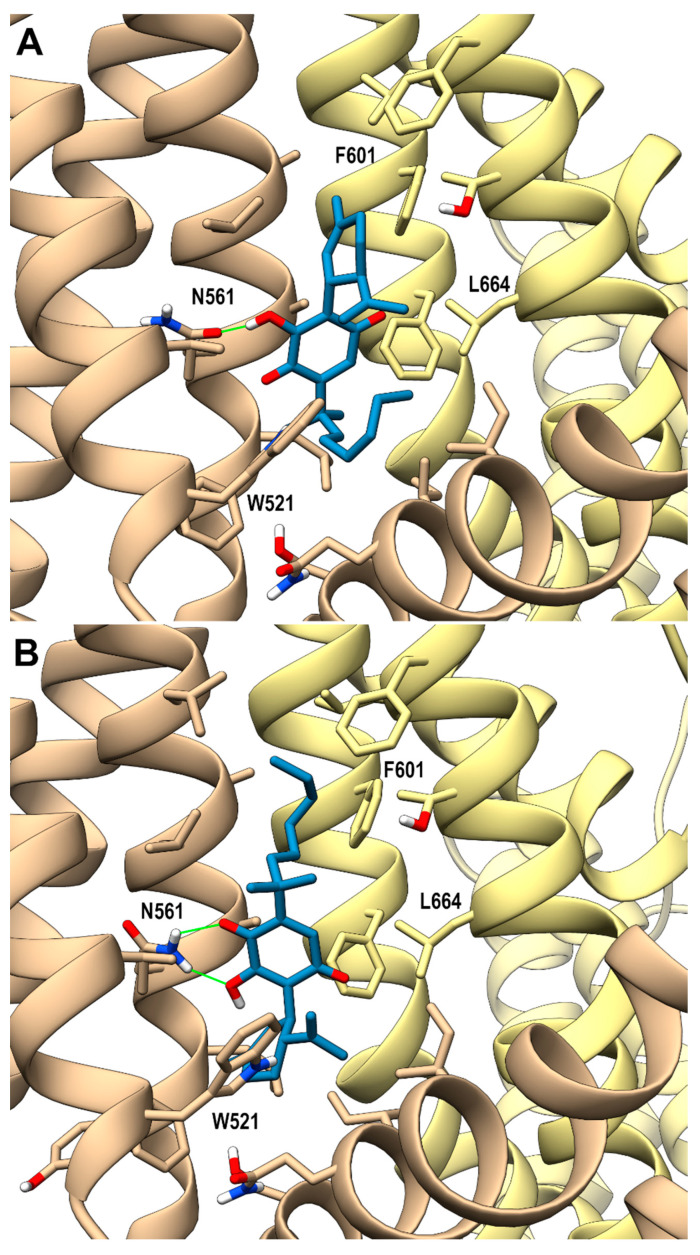
hTRPV3 in a complex with compound **5b** colored in steel blue (panel (**A**) shows the p-sI-A, panel (**B**) p-sI-B) at the vanilloid site (sI in the main text). A ribbon representation is used for the protein backbone and sticks for protein side chains of residues within 5 Å from the ligand, in a ball and stick representation. The two adjacent monomers are colored in tan and yellow. Carbon atoms are painted according to receptor subunits. H-bonds are shown as green sticks. Nitrogen, oxygen, and polar hydrogen atoms are painted blue, red, and white, respectively.

**Figure 3:**
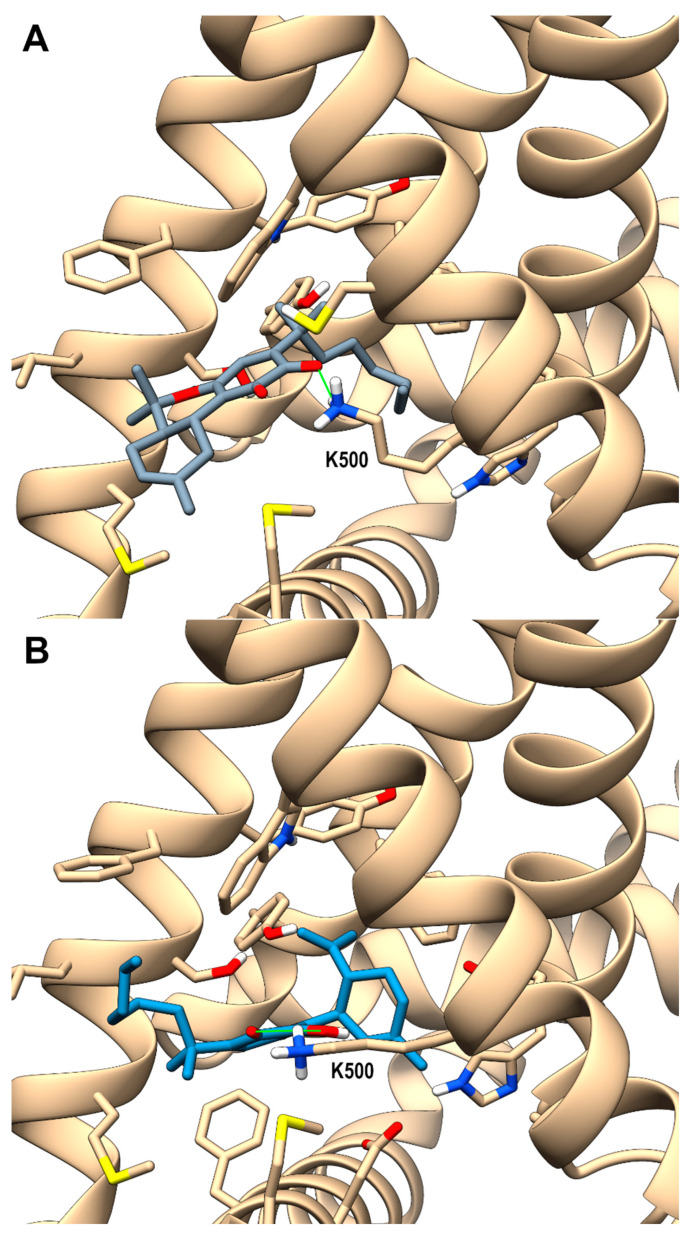
hTRPV3 in a complex with compound **6b** (panel (**A**), p-sII-A) colored in dark gray and **5b** colored in steel blue (panel (**B**)**,** p-sII-B) at site II (sII in the main text), corresponding to the cavity formed by the S1–4 bundle and the TRP domain of each monomer. A ribbon representation is used for the protein backbone and sticks for protein side chains of residues within 5 Å from the ligand, in a ball and stick representation. Carbon atoms are painted according to receptor subunits. H-bonds are shown as green sticks. Nitrogen, oxygen, and polar hydrogen atoms are painted blue, red, and white, respectively.

Looking at the activity profile of the investigated compounds, it emerges that the quinone group, while leaving substantially unaffected the activity of the α,α-DMH derivatives at TRPA1, increases the potency of all compounds at TRPV3 (see Table 1). Thus, in order to shed light on the binding modes of the most active compounds within the series, i.e., **5b** and **6b,** a docking study was conducted on this receptor channel, exploiting the recent availability of the cryo-em structure of TRPV3 in the complex with tetrahydrocannabivarin (THCV) (PDB id: 8V6L) [47]. The binding site of THCV, referred to hereafter as site I (sI), is hosted in the vanilloid pocket, located at the interface between two adjacent monomers: one monomer contributes with residues Trp521, Phe522, and Val525 on helix S3, Leu557, Ala560, Asn561, and Leu563 on helix S4, and Ile579 and Ile583 on the S4-S5 linker, while the adjacent monomer contributes with residues Phe597 and Phe601 on helix S5, Thr660, and Leu664 on helix S6. For both compounds, two main docking poses were identified: pose A (p-sI-A), which recapitulates the experimentally observed binding mode of THCV, and pose B (p-sI-B), flipped by 180° with respect to p-sI-A. However, according to the better value of the interaction energy calculated on the energy minimized complexes, only p-sI-A was selected as representative of **6b.** As shown in Figure 1, the ortho-quinone group is engaged in a bifurcate H-bond with Asn561 (S4) of one monomer, with the ligand being hosted in a pocket at the interface between the two monomers surrounded by aromatic and hydrophobic residues. Instead, both poses (shown in Figure 2) were selected as representative of compound **5b**, their values of interaction energy being very close (−56.4 vs. −57.7 kcal/mol, respectively). In both arrangements, compound **5b** is engaged in H-bond interactions with Asn561 (S4): in p-sI-A, it substantially overlaps the binding mode of compound **6b**, while in p-sI-B, the terpenoid ring is surrounded by aliphatic residues and stacks against Trp521, whereas the alkyl chain is hosted in the hydrophobic groove at the interface between the two monomers. Moreover, since the introduction of the quinone group increases the polarity of the pCBs, it is possible to hypothesize that they could also bind to additional sites, in particular to the binding pocket formed by the helix bundle S1–S4 and the TRP domain, that is lined by polar and aromatic residues. This site, hereafter termed site II (sII), is occupied either by a lipid molecule (in the TRPV3 structure in a complex with THCV, PDB id: 8V6L), or by ligands (2-APB, PDB id: 8V6N, or osthole, PDB id: 7RAU) [47,48]. In fact, although more polar than sI, sII is still compatible with the hydrophobic nature of pCBs, being located in the transmembrane region. Thus, the docking study was also extended to sII, where, as for sI, two main orientations, flipped by 180° to each other, were identified. On the basis of the interaction energy, the most favorable binding poses for compounds **6b** and **5b** are p-sII-A, with the alkyl chain hosted in the binding site, and the flipped orientation (p-sII-B), with the alkyl chain facing the membrane, respectively. As shown in Figure 3, both compounds are engaged in an H-bond interaction with Lys500 (S2), although with reverse orientations.

## 3. Discussion

Compounds **5b**–**9b**, which include *ortho*- and *para*-quinone dimethyl derivatives, were profiled against the TRP channels TRPV1-4, TRPM8, and TRPA1 (thermo-TRPs) and their activity compared to that of the corresponding non-quinone phyto-dimethyl derivatives **5a**–**9a** [36], to evaluate whether and to what extent the introduction of the quinone moiety on the resorcinol scaffold affects their efficacy, potency, and selectivity with this class of receptor channels.

The quinone derivatives **5b**–**9b** are inactive at both TRPV1 and TRPV4 (see Table 1), as already observed for their non-quinone counterparts **5a**–**9a** [36]. At TRPM8, the introduction of the quinone group in the dimethyl-heptyl series is detrimental to the activity, all compounds remaining inactive, with the only exceptions being **5b** (IC_50_ = 1.2 vs. 13.4 μM of **5a**) and **7b**, which, however, is less active than **7a** (IC_50_ = 14.9 vs. 9.2 μM). Instead, at TRPV2, all the compounds perform better than their non-quinone counterparts (Biomedicines ref.), as shown in Table 2, with an IC_50_ < 10 μM except for **7b**, which, however, is still more active than **7a** (IC_50_ = 23.7 vs. 39.4 μM). The activity as TRPA1 agonists/desensitizers within the series is substantially preserved with respect to the non-quinone DMH analogs, although some differences in the IC_50_ values can be observed: while the inhibitory potencies of the **7b** and **8b** derivatives are similar to **7a** and **8a** (IC_50_ = 1.1 vs. 1.7 μM, and 0.35 vs. 0.37 μM, respectively), those of **5b** and **9b** are slightly lower than **5a** and **9a** (IC_50_ = 3.5 vs. 0.51 and 15.1 vs. 3.2 μM, respectively) whereas **6b** performs slightly better than **6a** (IC_50_ = 1.8 vs. 6 μM). However, the most striking result arises from the activity profile at TRPV3. In fact, while the non-quinone counterparts are from weak (IC_50_ > 30 μM for **5a** and **7a**) to inactive (IC_50_ > 50 μM **8a** and **9a**) at this receptor channel, with the only exception of **6a** (IC_50_ = 2.1 μM), both **5b** and **6b** show a submicromolar activity as agonists/desensitizers and **7b** and **9b** an IC_50_ < 10 and <20 μM, respectively. From the structure−activity relationship analysis, it emerges that the methyl−cycloexenyl ring is critical for the highest activity, occurring in both **5b** and **6b** compounds, regardless of the position of the double bond. In fact, both the aromatization of the cycle (**9b**) and the lack of the cycloexenyl ring (**7b** and **8b**) are detrimental to the activity. However, **7b** performs better than both **8b** and **9b**, likely due to the greater flexibility of the isoprenoid chain, strongly oriented by the chromene scaffold in **8b** and fully constrained in **9b**. Docking studies showed that the cycloexenyl group closely interacts with the protein, while the quinone group promotes the formation of H-bond interactions at both sI, with Asn561, and sII, with Lys500. The latter additional site, not seen in the experimental structure of TRPV3 in a complex with THCV, could be favored by the increased polarity of the pCBs quinones in comparison with the natural ones, thus concurring in eliciting the stronger potency of the investigated compounds at this receptor. Lastly, the different isomerism at the quinone ring characterizing the most active compounds identified in the series characterized in the present study, i.e., **5b** (para) and **6b** (ortho), which exhibited a comparable efficacy/potency toward TRPV3, shows that the effects of this isomerism are strongly coupled/cooperative with those of the specific scaffold attached to the ring, as expected because of the conformational strains (strong for **5b**, almost total for **6b**) constraining their relative orientations. In conclusion, the introduction of a quinone group within the pCBs-DMH scaffold, while not detrimental to the activity toward TRPA1, increases the potency toward TRPV3 as desensitizing agents. The dual TRPA1/TRPV3 activity profile of the cannabinoid quinone series could be beneficial for chronic skin diseases sustained by inflammatory conditions, thus deserving further investigation in in vivo models, especially for a careful assessment of their potential toxicological profile related to the occurrence of a quinone moiety. Furthermore, our study adds further pieces of knowledge on the structural requirements needed to elicit a TRPV3 modulation, which in turn could contribute to the development of novel and potent TRPV3 ligands.

## 4. Materials and Methods

### 4.1. Synthesis

#### 4.1.1. General Experimental Procedures

IR spectra were recorded on an Avatar 370 FT-IR Techno-Nicolet apparatus. ^1^H (400 MHz) and ^13^C (100 MHz) NMR spectra were measured on a Bruker Avance 400 MHz spectrometer. Chemical shifts were referenced to the residual solvent signal (CDCl_3_: *δ*_H_ = 7.21, *δ*_C_ = 77.0). Low- and high-resolution electrospray ionization mass spectrometry (ESI-MS) data were determined on an LTQ OrbitrapXL (Thermo Scientific, Waltham, MA, USA) mass spectrometer. Reactions were monitored by thin-layer chromatography (TLC) on Merck 60 F254 (0.25 mm) plates, visualized by staining with 5% H_2_SO_4_ in EtOH and heating. Organic phases were dried with Na_2_SO_4_ before evaporation. Chemical reagents and solvents were purchased from Sigma-Aldrich and were used without further purification unless stated otherwise. Petroleum ether with a boiling point of 40–60 °C was used. Silica gel 60 (70–230 mesh) was used for gravity column chromatography (GCC). All starting α,α-DMH-cannabinoids were available from previous studies in the area [36,49]. Compounds **6b**, **8b** and **9b** were already available in our library from previous studies in the field [46].

#### 4.1.2. SIBX Oxidation

Reaction with DMH-CBG (**7a**) as representative: To a cooled (ice-bath) solution of DMH-CBG (**7a**, 200 mg, 0.533 mmol, Rf = 0.46 in petroleum ether-EtOAc 9:1 as eluant) in ethyl acetate (10 mL), SIBX (39 wt. %, 1.274 g, 1.775 mmol, 3.33 molar equiv.) was slowly added in small portions. The cooling bath was then removed and the suspension was stirred at room temperature for 18 h; the reaction mixture was then filtered over a pad of celite (diatomaceous earth). The filtration cake was then washed with EtOAc (10 mL), and the pooled filtrates were washed with saturated Na_2_S_2_O_3_ (4 × 15 mL) and next with brine. After drying and evaporation, the residue was purified by GCC on silica gel (10 g, petroleum ether–EtOAc 95:5 as eluant) to obtain a dark red solid identified as *p*-DMHCBGQ (**7b**, 109 mg, 52%, Rf = 0.78 in petroleum ether-EtOAc 9:1). Similar differences in Rf between reactants and reaction products were observed for the SIBX oxidation of **5a** to **5b**. Full characterization of compounds **5b** and **7b**, including ^1^H and ^13^C spectra (Figures S1–S4) are reported in Supplementary Materials.

### 4.2. Thermo-TRP Assay

The compounds were tested on HEK-293 cells either stably transfected with rat TRPA1, TRPV2, TRPV3, TRPV4, TRPM8 or human TRPV1 or not-transfected, by performing a continuous monitoring of the intracellular calcium ([Ca^2+^]_i_) elevation during the experiments, following the protocol already described [36,50]. On the day of the experiment, the cells were loaded for 1 h in the dark at room temperature with the fluorescent probe Fluo-4 AM (4 μM in DMSO containing 0.02% Pluronic F-127). The cells were rinsed, resuspended in Tyrode’s solution (145 mM NaCl, 2.5 mM KCl, 1.5 mM CaCl_2_, 1.2 mM MgCl_2_, 10 mM D-glucose, and 10 mM HEPES, pH 7.4), and transferred to a quartz cuvette of a spectrofluorimeter (PerkinElmer LS50B; λEX = 488 nm, λEM = 516 nm) equipped with a PTP-1 fluorescence Peltier system (PerkinElmer Life and Analytical Sciences, Waltham, MA, USA) under continuous stirring. Cell fluorescence before and after the addition of various concentrations of tested compounds was measured, normalizing the effects against the response to ionomycin (IM, 4 μM). The effect on [Ca^2+^]_i_ in non-transfected HEK-293 cells were used as the baseline and subtracted from the values obtained from the transfected ones. The efficacy of the agonists was determined by normalizing their effect to the maximum Ca^2+^ influx effect on [Ca^2+^]_i_ observed with application of 4μM ionomycin. The effects of TRPA1 agonists are expressed as a percentage of the effect obtained with 100 μM allyl isothiocyanate (AITC). In the TRPV3 assay, rat TRPV3-expressing HEK-293 cells were first sensitized with the structurally unrelated agonist 2-aminoethoxydiphenyl borate (100 μM). For the TRPM8, the experiments were carried out at 22 °C. The potency (EC_50_ values) is determined as the concentration required to produce half-maximal increases in [Ca^2+^]_i_. Antagonist/desensitizing behavior is evaluated against the agonist of the TRP analyzed by adding the compounds directly in the quartz cuvette 5 min before stimulation of cells with the agonist (allylisothiocyanate (100 μM) for TRPA1, capsaicin (0.1 μM) for TRPV1, cannabidiol (2 μM) for TRPV2, thymol (100 μM) for TRPV3, GSK1016790A (10 nM) for TRPV4, and icilin (0.25 μM) for TRPM8). The IC_50_ value is expressed as the concentration exerting a half-maximal inhibition of the agonist effect, taking as 100% the effect on [Ca^2+^]_i_ exerted by the agonist alone. A dose-response curve fitting (sigmoidal dose-response variable slope) and parameter estimation were performed with Graph-Pad Prism 8 (GraphPad Software Inc., San Diego, CA, USA). All determinations were performed at least in triplicate.

### 4.3. Molecular Docking

Ligands were built with UCSF Chimera 1.1736 [51] followed by initial energy minimization (EM) at the molecular mechanics level, using AM1-BC charges. The molecules were then fully optimized using the GAMESS program2021 R1 (2021Jun30) [52] at the Hartree–Fock level with the STO-3G basis set and subjected to HF/6-31G*/STO-3G single-point calculations to derive the partial atomic charges using the RESP procedure. Docking studies were performed with AutoDock 4.21 [53] using the cryo-em structure of human TRPV3 solved in complex with THCV (PDB id: 8V6L), with which the rat TRPV3 shares >94% of sequence identity. Both the protein and the ligands were processed with AutoDock Tools (ADT) package version 1.5.6rc1 [53] to merge non-polar hydrogens, calculate Gasteiger charges, and select rotatable sidechain bonds. Grid dimensions of 60 × 60 × 60 and 60 × 60 × 50, centered in site 1 and site 2, respectively, were generated with the program AutoGrid 4.2 included in Auto-dock 4.2 distribution, with a spacing of 0.375 Å. Docking runs were carried out by either keeping the whole protein fixed or allowing the rotation of selected residues (Phe522, Asn561, Leu563, and Ile579 for site 1 and Lys500 for site 2), following the docking protocol already described [36]. The docking poses were selected on the basis of binding energy value, cluster population, and visual inspection. Representative complexes for each ligand were completed by addition of all hydrogen atoms and subjected to energy minimization with Amber16 package [54], using a ff14SB force field for the protein, and gaff parameters for the ligand [55]. Interaction energy for each complex was calculated using NAMD plugin in VMD 1.9.4a53 [56].

## Figures and Tables

**Table 1:** Efficacy, potency, and inhibitory effect on TRPA1, TRPV1, TRPV4, and TRPM8 channels.

	TRPA1			TRPV1	TRPV4	TRPM8
	Efficacy (% AITC 100 μM)	Potency EC_50_ μM	IC_50_ ^a^ TRPA1 μM	IC_50_ ^b^ TRPV1 μM	IC_50_ ^c^ TRPV4 μM	IC_50_ ^d^ TRPM8 μM
**5b**	120.4 ± 3.0	5.2 ± 0.8	3.5 ± 0.20	>100	>100	1.2 ± 0.1
**5a** [36]	133.4 ± 5.3	0.40 ± 0.15	0.51 ± 0.03	>100	>100	13.4 ± 2.1
**6b**	147.3 ± 7.3	4.4 ± 1.25	1.8 ± 0.3	>100	>100	>100
**6a** [36]	124.2 ± 4.0	4.8 ± 1.1	6.0 ± 0.9	>100	>100	39.9 ± 6.9
**7b**	114.2 ± 4.1	1.1 ± 0.2	1.1 ± 0.1	>100	>50	14.9 ± 3.9
**7a** [36]	99.2 ± 4.5	9.1 ± 2.0	1.7 ± 0.15	>100	35.4 ± 3.2	9.2 ± 0.8
**8b**	111.2 ± 2.1	0.19 ± 0.03	0.35 ± 0.02	>100	>100	>100
**8a** [36]	120.4 ± 2.8	0.76 ± 0.12	0.32 ± 0.01	>100	>100	>100
**9b**	20.5	NA	15.1 ± 2.4	>100	>100	>100
**9a** [36]	132.3 ± 7.9	2.1 ± 0.9	3.2 ± 0.6	>100	>100	0.98 ± 0.12

Reference agonists: ^a^ AITC 100 μM; ^b^ Capsaicin 0.1 μM; ^c^ GSK1016790A 10 nM; ^d^ Icilin 0.25 μM.

**Table 2:** Efficacy, potency, and inhibitory effect on TRPV2 and TRPV3.

	TRPV2			TRPV3		
	Efficacy (% Ionomycin 4 μM)	Potency EC_50_ μM	IC_50_ ^a^ TRPV2 μM	Efficacy (% Ionomycin 4 μM)	Potency EC_50_ μM	IC_50_ ^b^ TRPV3 μM
**5b**	30.6 ± 6.5	12.2 ± 9.7	5.45 ± 0.5	64.3 ± 0.9	0.46 ± 0.02	0.76 ± 0.01
**5a [36]**	<10	NA	16.8± 0.2	15.8 ± 0.4	11.0 ± 1.1	32.6 ± 5.1
**6b**	78.1 ± 4.0	>50	8.5 ± 0.5	66.8 ± 2.2	0.50 ± 0.1	0.72 ± 0.08
**6a [36]**	<10	NA	45.8 ± 3.9	53.8 ± 1.6	0.14 ± 0.03	2.1 ± 0.6
**7b**	47.8 ± 1.4	30.5 ± 3.9	23.7 ± 2.9	51.1 ± 1.0	1.5 ± 0.2	8.6 ± 0.2
**7a [36]**	65.3 ± 0.7	9.8 ±0.6	39.4 ± 4.5	64.6 ± 4.8	39.7 ± 0.03	35.4 ± 3
**8b**	31.5 ± 2.9	>50	6.9 ± 0.1	37.55 ± 0.85	13.6 ± 1.8	38.3 ± 2.0
**8a [36]**	76.1 ± 0.6	>50	22.6 ± 0.2	41.6 ± 1.8	>50	>50
**9b**	23.4 ± 3.7	2.0 ± 1.6	5.2 ± 0.6	32.5 ± 0.65	1.15 ± 0.15	19.4 ± 3.5
**9a [36]**	10.5 ± 0.02	0.25 ± 0.03	>50	20.9 ± 3.0	41.4 ± 21.6	>100

Reference agonists: ^a^ CBD 2 μM; ^b^ Thymol 100 μM.

## Data Availability

All relevant data are within the paper and its Supplementary Materials.

## Supplementary Materials

The following supporting information can be downloaded at: https://www.mdpi.com/article/10.3390/ijms26062682/s1.
