# Supplementary material for: The Impact of a Quinone Scaffold on Thermo-TRPs Modulation by Dimethylheptyl Phytocannabinoids
Source: Int J Mol Sci. 2025 Mar 17;26(6):2682. doi: 10.3390/ijms26062682 (PMC11942486; doi:10.3390/ijms26062682)
Supplement: Supplementary file 1 [file ijms-26-02682-s001.zip › ijms-3526947-supplementary.pdf]

# The Impact of a Quinone Scaffold on Thermo-TRPs Modulation by Dimethylheptyl Phytocannabinoids

**Aniello Schiano Moriello [1,2,†], Aurora Bossoni [3,†], Daiana Mattoteia [3], Diego Caprioglio [3], Alberto Minassi [3], Giovanni Appendino [3], Luciano De Petrocellis [1], Pietro Amodeo [1] and Rosa Maria Vitale [1,*]**

[1] Institute of Biomolecular Chemistry, National Research Council (ICB-CNR), Via Campi Flegrei 34, 80078 Pozzuoli, Italy; aniello.schianomoriello@icb.cnr.it (A.S.M.); luciano.depetrocellis@icb.cnr.it (L.D.P.); pamodeo@icb.cnr.it (P.A.)
[2] Epitech Group SpA, Saccolongo, 35100 Padova, Italy
[3] Dipartimento di Scienze del Farmaco, Università del Piemonte Orientale, Largo Donegani 2, 28100 Novara, Italy; aurora.bossoni@unipo.it (A.B.); daiana.mattoteia@uniupo.it (D.M.); diego.caprioglio@unipo.it (D.C.); alberto.minassi@unipo.it (A.M.); giovanni.appendino@unipo.it (G.A.)
* Correspondence: rmvitale@icb.cnr.it
† These authors contributed equally to this work.

## Supplementary Information

***para*-3'-Depentyl-3'-(α,α-dimethylheptyl)cannabidiol quinone (*p*-DMHCBDQ, 5b):** dark red solid, 56% yield, Rf= 0.61 in petroleum ether-EtOAc 95:5. IR $v_{max}$ (KBr disc): 2970, 2918, 2852, 1674, 1136, 1127, 1107, 798 $cm^{-1}$. $^1H$ NMR (400 MHz, Chloroform-*d*) δ $^1H$ NMR (400 MHz, Chloroform-*d*) δ 7.20 (s, 1H), 6.40 (s, 1H), 5.18 (s, 1H), 4.71–4.50 (m, 2H), 3.74 (dd, *J* = 10.8, 2.1 Hz, 1H), 2.77 (ddd, *J* = 12.1, 10.7, 2.9 Hz, 1H), 2.31–2.19 (m, 1H), 2.02 (dd, *J* = 17.5, 5.3 Hz, 1H), 1.85 –1.63 (m, 7H), 1.59 (s, 3H), 1.45–1.17 (m, 12H), 1.08-0.97 (m, 2H), 0.88 (t, *J* = 6.9 Hz, 3H). $^{13}C$ NMR (100 MHz, $CDCl_3$) δ 187.25, 183.49, 151.73, 150.07, 148.43, 135.37, 133.94, 129.50, 122.40, 121.73, 110.62, 44.63, 40.61, 38.17, 35.69, 31.67, 30.46, 29.74, 28.78, 27.27, 27.24, 24.99, 23.45, 22.57, 18.70, 14.05. HR ESI-MS *m/z* 385.5672 $[M + H]^+$, calcd for $C_{25}H_{37}O_3$, 385.5680.

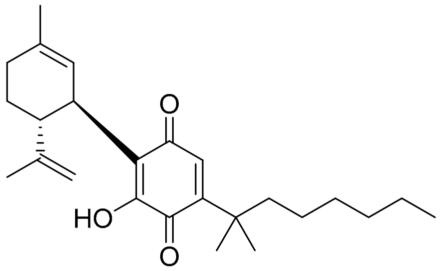


***para*-3'-Depentyl-3'-(α,α-dimethylheptyl)cannabigerol quinone (*p*-DMHCBGQ, 7b):** dark red solid, 52% yield, Rf= 0.78 in petroleum ether-EtOAc 9:1. IR $v_{max}$ (KBr disc): 3257, 2948, 2870, 1642, 1633 , 1361, 1317, 1176, 1169, 584 $cm^{-1}$. $^1H$ NMR (400 MHz, Chloroform-*d*) δ 7.22 (bs, OH, 1H), 6.46 (s, 1H), 5.16 (t, *J* = 7.4 Hz, 1H), 5.05 (t, *J* = 7.2 Hz, 1H), 3.14 (d, *J* = 7.4 Hz, 2H), 2.09-1.93 (m, 4H), 1.78-1.68 (m, 2H), 1.75 (s, 3H), 1.65 (s, 3H), 1.58 (s, 3H), 1.30-1.17 (m, 12H), 1.09-0.99 (m, 2H), 0.86 (t, *J* = 6.8 Hz, 3H). $^{13}C$ NMR (100 MHz, $CDCl_3$) δ 187.60, 183.58, 151.36, 150.52, 137.06, 134.97, 131.34, 124.20, 120.26, 119.58, 119.04, 40.65, 39.70, 38.26, 31.66, 29.79, 27.34, 26.57, 25.67, 25.02, 22.61, 21.79, 17.65, 16.16, 14.03. HR ESI-MS *m/z* 387.2895 $[M + H]^+$, calcd for $C_{25}H_{39}O_3$, 387.2899.

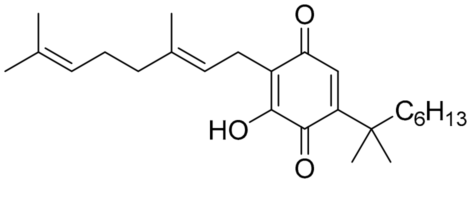

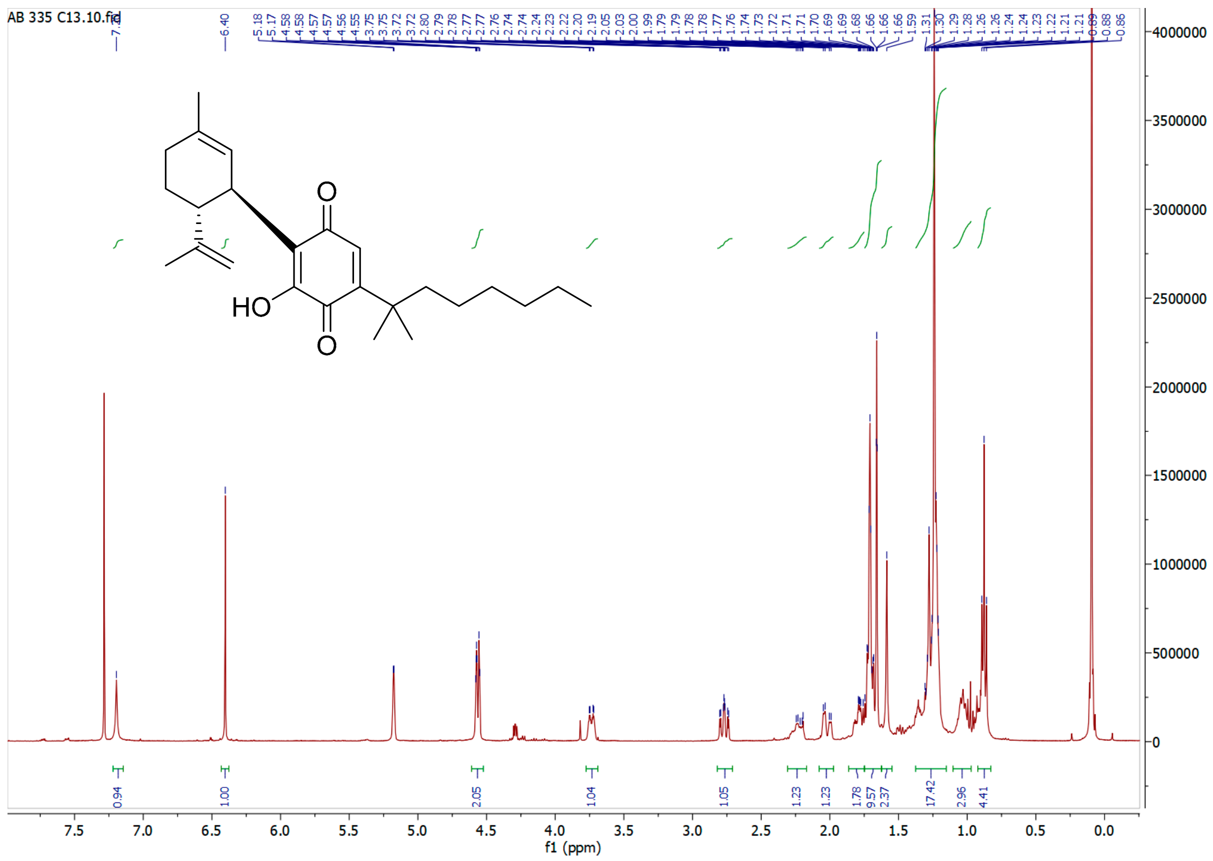

**Figure S1.** $^{1}H$ NMR spectrum (400 MHz) of compound **5b** in $CDCl_3$.

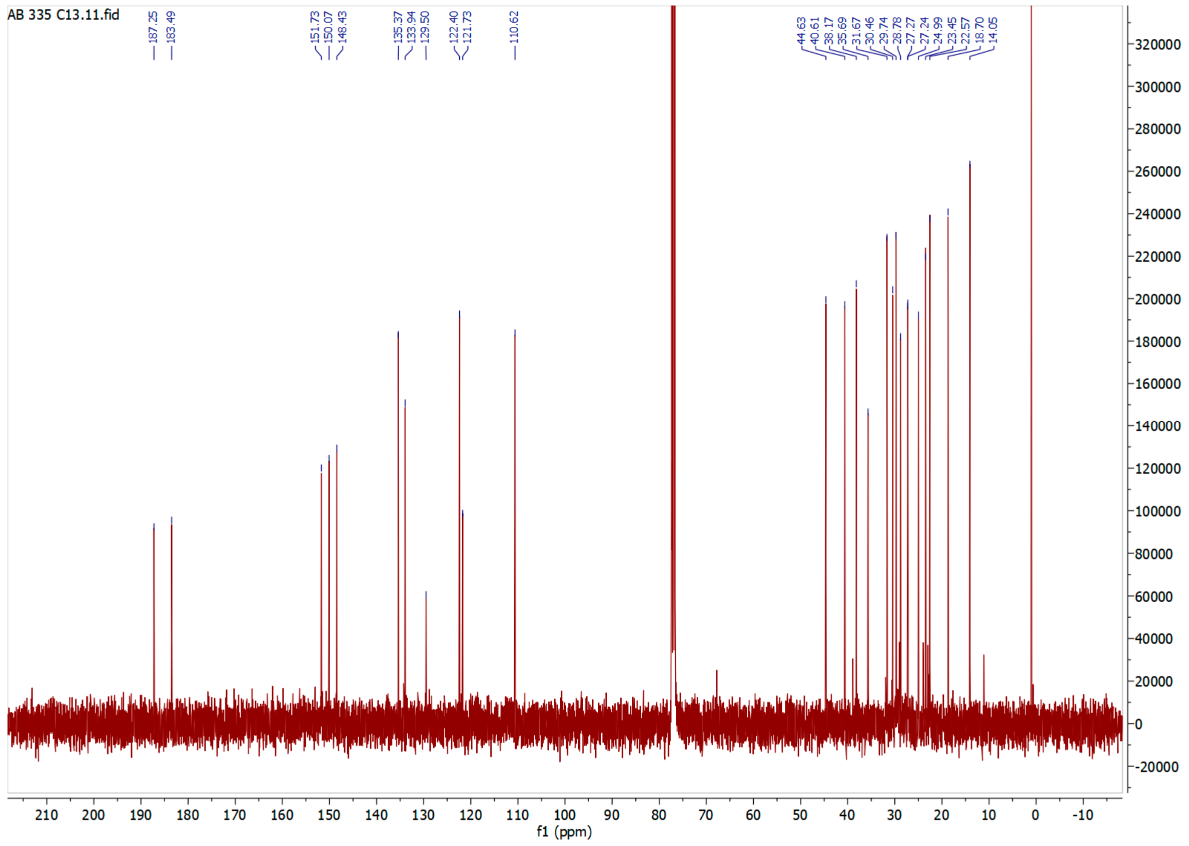

**Figure S2.** $^{13}C$ NMR spectrum (100 MHz) of compound **5b** in $CDCl_3$.

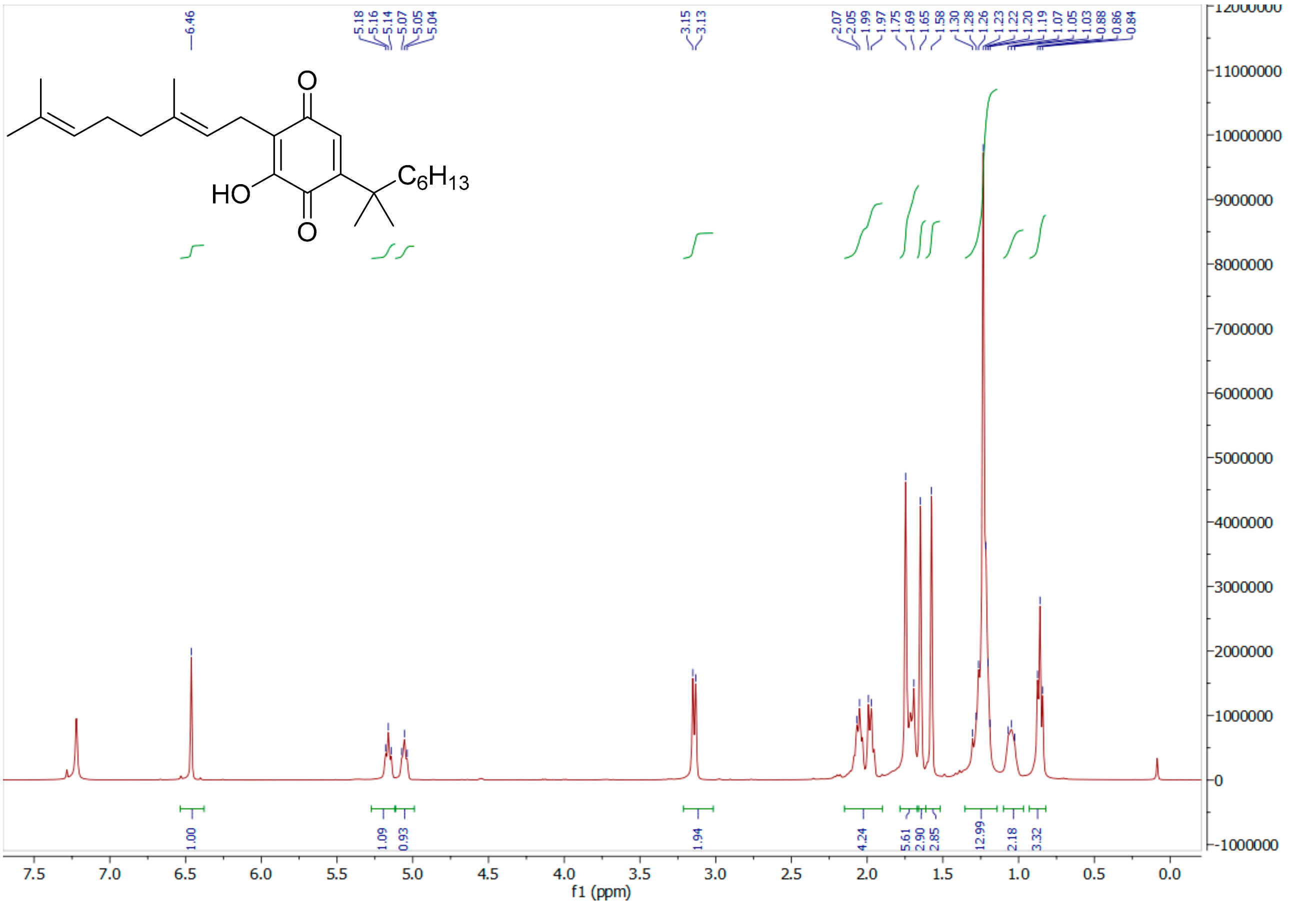


**Figure S3.** $^{1}$H NMR spectrum (400 MHz) of compound **7b** in $CDCl_3$.

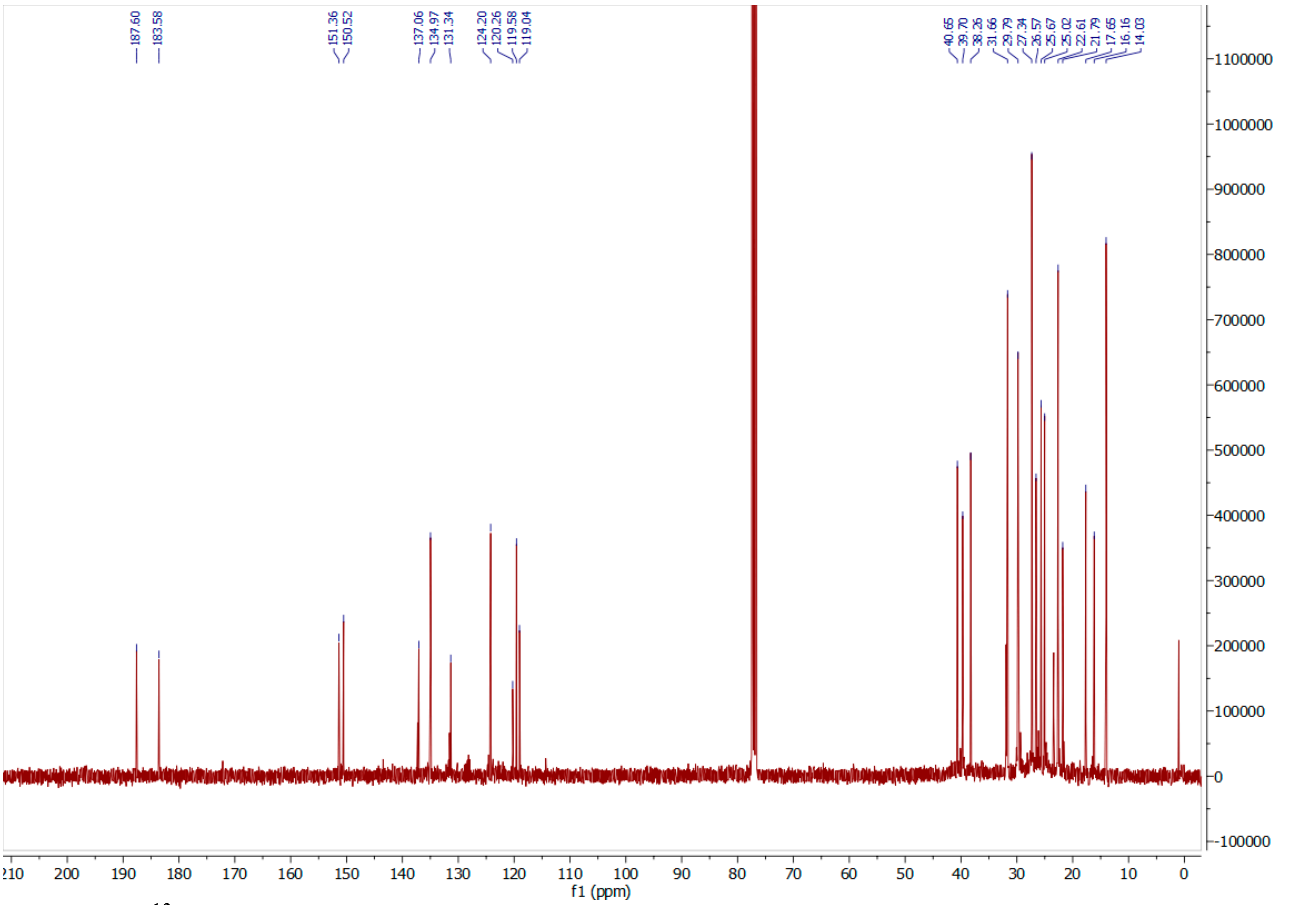


**Figure S4.** $^{13}$C NMR spectrum (100 MHz) of compound **7b** in $CDCl_3$.
